# Efficacy of a physical activity programme combining individualized aerobic exercise and coaching to improve physical fitness in neuromuscular diseases (I’M FINE): study protocol of a randomized controlled trial

**DOI:** 10.1186/s12883-020-01725-0

**Published:** 2020-05-13

**Authors:** Sander Oorschot, Merel A. Brehm, Annerieke C. van Groenestijn, Fieke S. Koopman, Camiel Verhamme, Filip Eftimov, Judith G. M. Jelsma, Harald T. Jorstad, Frans Nollet, Eric L. Voorn

**Affiliations:** 1 Department of Rehabilitation, Amsterdam Movement Sciences, Amsterdam UMC, University of Amsterdam, Meibergdreef 9, Amsterdam, The Netherlands; 2 Department of Neurology, Amsterdam Neuroscience, Amsterdam UMC, University of Amsterdam, Meibergdreef 9, Amsterdam, The Netherlands; 3 Department of Public and Occupational Health, Amsterdam UMC, VU University Medical Center, de Boelelaan 1118, Amsterdam, The Netherlands; 4 Department of Cardiology, Amsterdam Movement Sciences, Amsterdam UMC, University of Amsterdam, Meibergdreef 9, Amsterdam, The Netherlands

**Keywords:** Neuromuscular diseases, Physical fitness, Active lifestyle, Aerobic exercise, Coaching, Motivational interviewing

## Abstract

**Background:**

In individuals with neuromuscular diseases (NMD), symptoms of muscle weakness, fatigue and pain may limit physical activity. Inactivity leads to reduced physical fitness, which further complicates daily life functioning. Due to inconclusive evidence regarding exercise in NMD, the optimal training approach and strategies to preserve an active lifestyle remain to be determined. The physical activity programme I’M FINE, consisting of individualized aerobic exercise to improve physical fitness and coaching to preserve an active lifestyle, was therefore developed. The primary objective of this study will be to evaluate the efficacy of the I’M FINE programme in terms of improved physical fitness in individuals with slowly progressive NMD, compared to usual care.

**Methods:**

A multicentre, assessor-blinded, two armed, randomized controlled trial will be conducted in a sample of 90 individuals with slowly progressive NMD. Participants motivated to improve their reduced physical fitness will be randomized (ratio 1:1) to the I’M FINE intervention or usual care. The I’M FINE intervention consists of a six-month physical activity programme, including individualized home-based aerobic exercise to improve physical fitness (i.e. peak oxygen uptake), and motivational interviewing coaching (e.g. goal setting, self-management) to adopt and preserve an active lifestyle. Measurements will be performed at baseline, post-intervention, and at 12- and 18-months follow-up. The primary outcome is peak oxygen uptake (VO_2_ peak) directly post intervention. Main secondary outcomes are physical capacity, muscle strength, self-efficacy, daily activity, quality of life and markers of metabolic syndrome. The primary analysis compares change in VO_2_ peak post-intervention between the intervention and usual care group, with analysis of covariance.

**Discussion:**

The I’M FINE study will provide evidence regarding the efficacy of a physical activity intervention on the physical fitness and active lifestyle over the short- and long-term in individuals with slowly progressive NMD. These outcomes could potentially improve the (inter)national guidelines for efficacy of aerobic exercise programmes and provide insight in achieving a more active lifestyle in NMD.

**Trial registration:**

(5/11/2018): Netherlands Trial Register NTR7609 (retrospectively registered), https://www.trialregister.nl/trial/7344. However, the Ethics Review Committee of the Amsterdam Medical Center (AMC) approved the study protocol on 7/11/2017. No adjustments were made to the approved study protocol before the first participant enrolment and registration. Registration was done after the second participant enrolment and the information in the register corresponds one on one with the approved study protocol.

## Background

Promotion of physical activity is a central component in the prevention and treatment of numerous diseases, maintenance of functional independence, and improvement of general well-being and life satisfaction [1–3]. However, in slowly progressive neuromuscular diseases (NMD), common symptoms like fatigue [4], poor endurance capacity [5], and pain [6] lead to increased difficulty when engaging in physical activity, leading to reduced physical fitness. Over 40% of individuals with slowly progressive NMD experience ‘difficulty exercising’ as main problem impacting daily life functioning [7, 8]. In turn, reduced physical fitness and a sedentary lifestyle negatively affect general health and daily life functioning, which is substantiated by the high prevalence of metabolic syndrome (55%) in this population [9–11]. Altogether, the vicious cycle of reduced physical fitness in slowly progressive NMD may be due to the disease itself (e.g. reduced muscle mass), which is irreversible, or to inactivity, which is reversible. In this study, we focus on reduced physical fitness due to inactivity.

Breaking the vicious cycle of inactivity and reduced physical fitness by means of aerobic exercise is a central goal of rehabilitation management [12]. Although some studies in slowly progressive NMD demonstrated positive short-term effects of aerobic exercise on physical fitness, other studies reported negative results [13–15], thus the overall evidence is inconclusive. Most exercise studies in NMD used conventional programmes originally designed for able-bodied individuals. In these programmes, intensity prescription was based on estimated fitness levels, rather than on the individuals’ actual fitness level. This lack of individualization, which leads to relatively high intensity levels, makes it difficult for individuals with NMD to adhere to their programme and likely contributes to the high dropout rates reported [16–18]. Polarized aerobic exercise appears to be a promising alternative for conventional training in this population. In this type of training, approximately 75% of total training volume is performed at low intensities and approximately 25% performed at high intensities [19]. This new approach was derived from training schedules of elite endurance athletes, and has been successfully applied in various diseases such as cancer [20] and obesity [21].

To sustain the health benefits associated with aerobic exercise, approaches to ensure continuation of exercise behaviour after completion of the training programme have to be considered, especially in chronic diseases such as NMD [22]. This requires behavioural and/or technological solutions including strategies like goal setting, self-monitoring, and feedback. However, few studies on exercise interventions among NMD have focused on the sustainability of acquired results of exercise programmes [23]. Based on other studies of chronic health conditions such as obesity [24] and heart failure [25], motivational interviewing (MI) seems to be a promising basis for implementation of a coaching programme in interventions to increase physical activity within the NMD population [26–29].

To our knowledge, no previous studies in slowly progressive NMD have evaluated the combined effects of aerobic exercise and coaching to increase and preserve physical fitness. Therefore, our research group developed a physical activity programme for individuals with slowly progressive NMD, called IMproving FItness in NEuromuscular diseases (I’M FINE). Key components are: 1) comprehensive assessment of the participants’ actual physical fitness and physical activity level, 2) individualized polarized aerobic exercise to improve physical fitness, and 3) motivational interviewing coaching to attain and preserve an active lifestyle. The primary objective of this study is to evaluate the efficacy of the six-month I’M FINE intervention on physical fitness in individuals with slowly progressive NMD, in comparison with usual care. Secondary objectives are to evaluate the longer-term (6 and 12 months after intervention) effects on physical fitness (sustainability), and to evaluate effects on daily activity, quality of life, perceived physical functioning, muscle function, markers of metabolic syndrome and self-efficacy. Furthermore, the underlying mechanisms of improved physical fitness and daily activity in individuals with slowly progressive NMD will be studied.

## Methods

### Study design

**Fig. 1:**
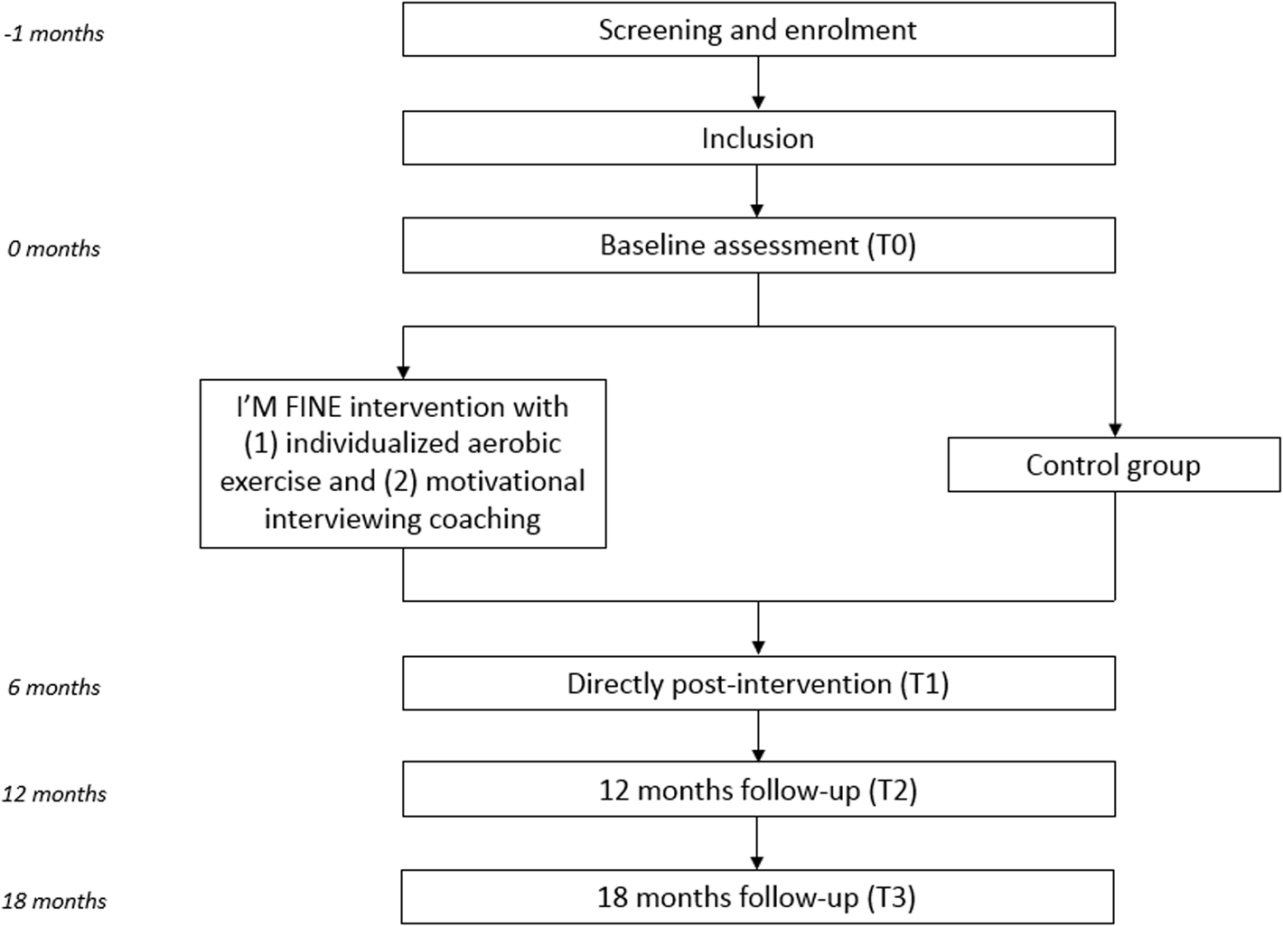
Schematic representation of the study design

This is a multicentre, assessor-blinded, two-armed, randomized controlled trial (RCT), with measurements at baseline (T0), directly after intervention (T1) and at 12 (T2) and 18 months (T3) follow-up (Fig. 1). The I’M FINE study protocol was written in accordance with the Standard Protocol Items: Recommendations for Interventional Trials (SPIRIT) checklist [30], as included in appendix 1.

### Study population

The study population consists of individuals with slowly progressive NMD, focusing on prior poliomyelitis and Post-Polio Syndrome (PPS), Charcot-Marie-Tooth disease (CMT), and other slowly progressive NMD. Participants will be recruited from six participating hospitals and rehabilitation centres and through the nationwide patient organization for NMD ‘Dutch Association for Neuromuscular Diseases’. The participating centres are Amsterdam University Medical Center, location AMC (Amsterdam), University Medical Center Utrecht (Utrecht), Rehabilitation Center Klimmendaal (Arnhem), hospital Sint Maartenskliniek (Nijmegen) and Merem Medical Rehabilitation (Almere and Hilversum). Potentially eligible individuals will receive an information letter. Subsequently, during a phone call, current attitudes and beliefs about exercising in NMD, actual physical fitness and activity levels, and barriers and facilitators to physical activity will be discussed. Eligible individuals willing to participate will be invited for a screening visit. After providing informed consent, individuals will undergo baseline assessment to confirm definitive eligibility for inclusion. A participant must meet all the following inclusion criteria:

- Diagnosed with prior poliomyelitis (confirmed by signs of residual weakness and atrophy of muscles on neuromuscular examination, and with electromyography); PPS (according to the March of Dimes criteria [31]); CMT (confirmed by DNA testing or polyneuropathy compatible with CMT and positive family history); or other slowly progressive NMD (with no effective drug therapy).
- Motivated to improve a reduced physical fitness level.
- Aged ≥18 years.

And not fulfil any of the following exclusion criteria:

- Contraindication for physical activity according to the American College of Sports Medicine (ACSM) guidelines [32].
- Unable to follow verbal or written instructions.
- Insufficient competence in the Dutch language.
- Engaged in an exercise programme (planned, structured, and repetitive physical activity performed at sufficient intensity to improve or maintain physical fitness) for a period longer than 4 weeks in the past 6 months.

### Randomization and blinding

After baseline assessment and study enrolment, participants will be randomized to the intervention or control group. Randomization will be stratified for diagnosis and treatment centre. We aim for equal group sizes of the three different diagnoses. The randomization scheme will be computer-generated in a Castor EDC database (Castor EDC, Amsterdam, The Netherlands), which uses random blocks of sequences with variable block sizes of two and four. The study coordinator, who is not involved in outcome assessments, will perform the randomization. Independent investigators blinded to group allocation will assess outcomes. Participants cannot be blinded for group allocation, but will be instructed not to reveal their group allocation to the investigators. Analyses will be performed blinded for group allocation.

### Intervention

The I’M FINE intervention consists of individualized aerobic exercise and motivational interviewing coaching.

#### Individualized aerobic exercise

Physiotherapists experienced in treating NMD will supervise the individualized aerobic exercise training. They will receive a one-day basic training, including general information about aerobic exercise in this population, training principles, and study-specific information, such as the session manual and exercise testing.

**Fig. 2:**
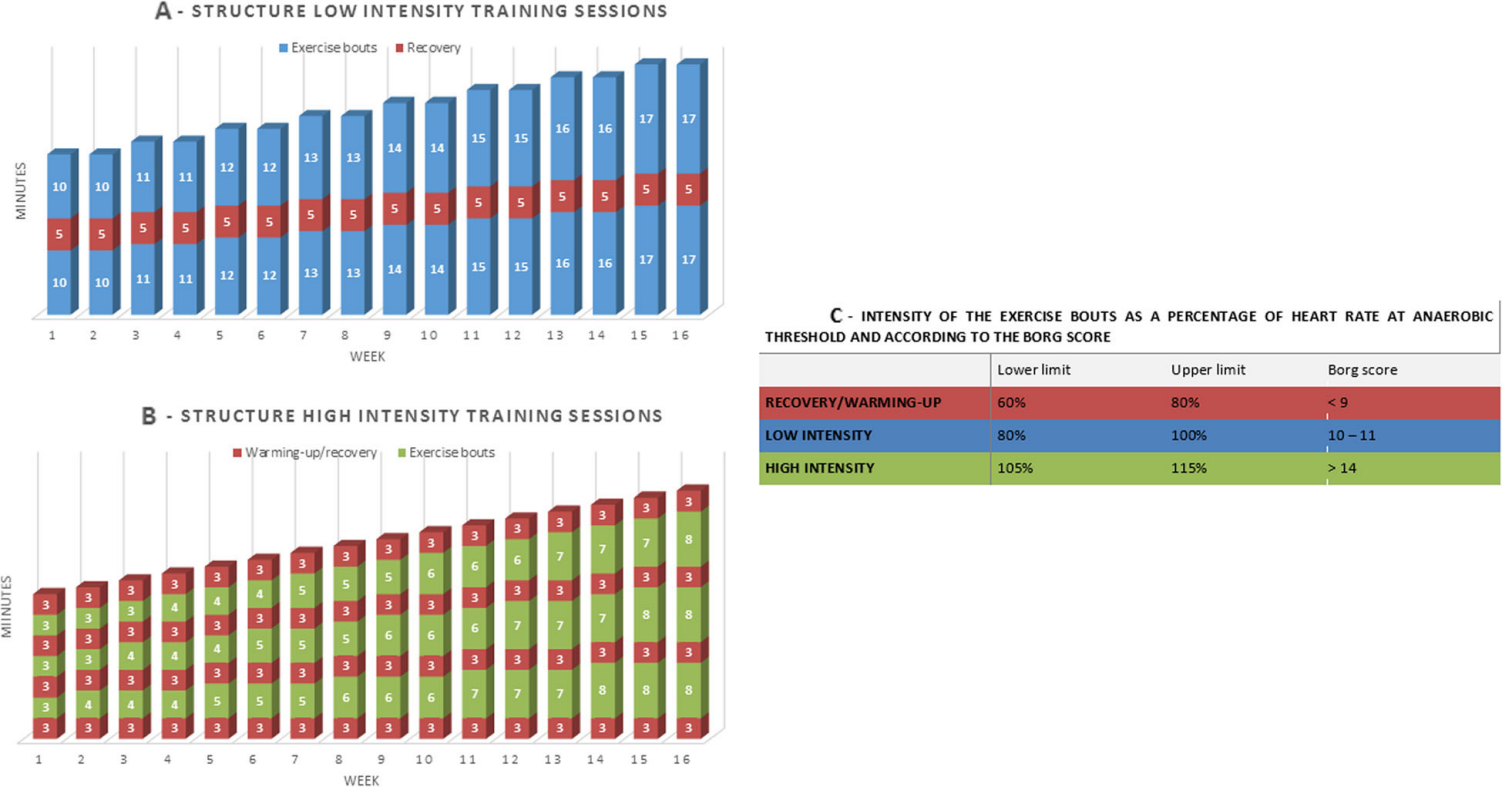
Structure and intensity of the training sessions. Figure 2a visualizes the structure of the low intensity training sessions. The blue blocks represent the low intensity exercise bouts, interspersed by the recovery bouts in red. Figure 2b visualizes the structure of the high intensity training sessions. The green blocks represent the high intensity exercise bouts, interspersed by the recovery bouts in red. Figure 2c represents the intensities of the different training sessions and recovery/warming-up

Individualized aerobic exercise consists of a 16-week polarized home-based programme, including two low-intensity sessions below the anaerobic threshold (AT) and one high-intensity session above the AT per week. Supervision consists of six individual face-to-face sessions and three telephone sessions. The exercise sessions consist of multiple exercise bouts per session, which will be gradually increased in duration (Fig. 2). Target heart rate ranges per exercise bout (i.e. low intensity, high intensity and recovery) are based on the AT (Fig. 2c), which will be determined from the maximal exercise test during baseline assessment and evaluated after 8 weeks of training [33]. If the AT cannot be determined from the maximal exercise test or a participant cannot train within the target heart rate ranges (e.g. in case of beta-blocking agents), the training programme will be based on the Borg scale [34].

Training sessions will be performed on a stationary ergometer. Participants will be supplied with the ReVi-app (Amsterdam UMC, Amsterdam, the Netherlands) and a heart rate monitor chest strap (Polar H10, Polar Electro, Kempele, Finland), which connect to each other to continuously monitor heart rate during training. The ReVi-app was specifically designed for the I’M FINE aerobic exercise programme and will be programmed with the participant’s target heart rate ranges. It provides real-time auditory feedback to support participants in maintaining their heart rate within the target range. In addition, participants will register their perceived exertion on the Borg Scale (range 6–20) after every exercise bout [35]. All data collected via the ReVi-app are displayed and stored in a digital dashboard. Physiotherapists will use this dashboard to monitor adherence and possible physical complaints (e.g. muscle soreness, cramps). If necessary, physiotherapists will make and register adjustments to training schedules.

#### Motivational interviewing coaching

Parallel to the individualized aerobic exercise, participants will receive motivational interviewing coaching consisting of eight individual face-to-face sessions and three telephone sessions, focused on identification of individual beliefs and aims, to promote a physically active lifestyle. Motivational interviewing is a “collaborative, goal-oriented style of communication with particular attention to language of change. It is designed to strengthen personal motivation for and commitment to specific goals by eliciting and exploring the person’s own reasons for change within an atmosphere of acceptance and compassion” [36]. Supervising practitioners will be provided with a manual containing session contents. Core elements are (1): education on fitness (2), goal setting (3), personal coaching, and (4) feedback on daily activity (Table 1).

**Table 1:** Core elements of motivational interviewing coaching

(1) Education on physical fitness in NMD: Participants receive specific strategies to promote behavioural change. These strategies include: education about the health benefits of physical activity, advice about activities that are suitable for individuals with NMD, education about training principles and polarized training, identifying and overcoming any perceived barriers to participation in physical activity, and recruiting social support from spouses, friends or other NMD individuals.	
(2) Goal setting: Participants set short- and long-term goals regarding activity and participation levels. SMART goals (specific, measurable, acceptable, realistic, timeline) are formulated in a systematic way.	
(3) Personal coaching: During the coaching sessions, the practitioner guides participants towards a more active lifestyle by integrating physical activity into daily life.	
(4) Feedback on daily activity: Participants will receive a FITBIT Flex (Fitbit Inc., San Francisco, CA), which provides feedback on the level of physical activity during daily life.	

Supervising practitioners (occupational therapists or movement teachers) have followed a basic course in MI [34, 35], and will participate in a one-day MI refresher course. To optimize MI coaching quality, an audio recording of a coaching session will be used to provide feedback from an experienced MI-assessor, who will score this according to the Motivational Interviewing Treatment Integrity (MITI) scoring list, version 4.2.1 [36].

### Usual care

Participants are allowed usual care. Usual care may include use of assistive devices, orthoses, regular physical therapy, and medication. Participants will not be restricted in their activities. Co-interventions will be monitored throughout the study.

### Outcomes

Outcome measures for this study are presented in Table 2. All outcomes will be collected and entered into a Castor EDC database by trained investigators. Outcomes will be assessed at baseline (T0), directly post-intervention (T1) and 12 months (T2) and 18 months (T3) follow-up.

**Table 2:** Outcome measures and assessment methods

		Visit			
		T0	T1	T2	T3
**Primary outcomes**	**Method**				
[1] *Physical fitness*	Maximal exercise test on bicycle ergometer or arm ergometer	X	X	X	X
**Secondary outcomes**					
[2] *Daily activity*	Heart rate monitoring and accelerometer	X	X	X	X
[3] *Health-related quality of life*	SF-36 questionnaire	X	X	X	X
[4] *Perceived physical functioning*	ACTIVLIM questionnaire	X	X	X	X
[5] *Muscle strength*	Fixed dynamometry	X	X	X	X
[6] *Markers of metabolic syndrome and muscle damage*	Blood analysis, waist circumference, blood pressure	X	X	X	X
[7] *Self-efficacy*	Self-efficacy scale	X	X	X	X
[8] *Physical capacity*	6-min walk test or 6-min push test	X	X	X	X
**Other**					
Demographic variables (age, gender, education, ethnicity, socioeconomic status)	Questionnaire	X			
Diagnosis and medical history	Questionnaire and medical record	X			

Abbreviations: T0; baseline assessment, T1; directly post-intervention, T2; 12 months follow-up, T3; 18 months follow-up, SF-36; Short Form 36–item Health Survey

#### Primary outcome - physical fitness

The primary outcome is the change in peak oxygen uptake (VO_2peak_). VO_2peak_ is considered the gold standard for physical fitness and recommended as primary outcome of exercise studies in NMD [14]. VO_2peak_ is measured during a maximal incremental exercise test on a bicycle or arm ergometer (Lode Excalibur, Groningen, The Netherlands) and breath-by-breath respiratory gas exchange will be measured with MasterScreen CPX (CareFusion, Hoechberg, Germany).

First, respiratory functions at rest are assessed, including forced vital capacity (FVC), forced expiratory volume in 1 s (FEV1), inspiratory capacity (IC) and maximal voluntary ventilation (MVV) [37, 38]. The exercise test will be executed according to international guidelines concerning standardization and will be supervised by trained researchers. The electrocardiogram and blood pressure are monitored during the test [39]. After a three-minute rest period to measure resting metabolism, the test starts with 3 minutes of unloaded (arm)cycling, followed by a ramp protocol with 5–20 W/minute continuous increments in workload, depending on the participants’ physical fitness level. Stopping criteria are: VO_2_ plateau, exhaustion, pedal frequency dropping below 50 rpm (RPM), and/or participant meeting the ACSM stop criteria [32].

#### Secondary outcomes

##### Daily activity

Daily physical activity will be measured using heart rate monitoring (Polar Electro, Kempele, Finland) during seven consecutive days in daily life to establish total time per day spent in low, moderate and vigorous intensity activities. An accelerometer (ActiGraph GT3X+, Health One Technology, Fort Walton Beach, FL) will be used to determine total daily step count.

##### Health-related quality of life

Health-related quality of life will be assessed using the Dutch validated version of the Short Form 36–item Health Survey (SF36). The physical health component score (PCS) and mental health component score (MCS) will be calculated [40].

##### Perceived physical functioning

Perceived physical functioning will be assessed with the originally developed and validated Dutch ACTIVLIM questionnaire, consisting of 22 daily activities for which perceived difficulty in performing the activity is scored on a scale with the options: impossible, difficult, easy or a ‘?’ [41]. With an online Rasch model, the raw scores will be converted into a linear measure of the participants’ perception of difficulty in performing activities of daily living [42].

##### Muscle strength

Muscle strength, quantified as maximal voluntary torque (MVT) will be assessed isometrically with a fixed dynamometer (Biodex System 4, New York, USA). Depending on the selected training mode, either the upper (elbow flexors and/or shoulder abductors) or lower extremity (knee extensors and/or plantar flexors) muscles will be measured. Only muscle groups with scores > 3 on the Medical Research Council (MRC) scale [43] will be measured. Three repetitions will be performed and peak torque in Newton-meters (Nm) used for analyses.

##### Markers for metabolic syndrome and muscle damage

The blood lipids total cholesterol, high density lipoprotein (HDL), low density lipoprotein (LDL), very low density lipoprotein (VLDL) and triglycerides, together with glucose, will be assessed from blood samples in a fasted state. Waist circumference will be recorded as the mean of two measurements with a SECA 201 device (Seca GmBH & Co Kg, Hamburg, Germany) and resting blood pressure as the mean of two measurements with the Datascope DUO^tm^ (Datascope Corp. New Jersey, USA). The presence of metabolic syndrome is defined as meeting three out of five criteria: triglyceride level ≥ 1.7 mmol/l; HDL ≤ 1.04 mmol/l for men or ≤ 1.29 mmol/l for women; fasting glucose ≥6.1 mmol/l; systolic blood pressure > 130 mmHg or diastolic blood pressure > 85 mmHg; or waist circumference > 102 cm for men or > 88 cm for women [44]. Furthermore, creatine kinase (CK) will be assessed as an indicator of muscle damage.

##### Self-efficacy

A Dutch translated version of the Self-Efficacy for Physical Activity (SEPA) scale will be used to determine self-efficacy [45]. This scale assesses participant’s confidence with regard to engaging in exercise in the presence of the barriers: feeling tired, bad mood, no time, on holiday or want to be active outside, but the weather is bad. Items are rated on a 5-point Likert scale (1 = absolutely no confidence, 5 = completely confident) and will translate to a total score for self-efficacy. The reliability and validity of the SEPA scale has been confirmed in various populations [46–48].

##### Physical capacity

The total distance covered at self-selected comfortable speed and oxygen consumption will be determined with the 6-min walk test (6MWT) or 6-min push test (6MPT) in case participants are wheelchair bound [49, 50]. During the test, breath-by-breath VO_2_ and VCO_2_ are measured with the K5 portable gas analysis system (Cosmed, Rome, Italy). The mean steady state VO_2_ and VCO_2_ (both in ml/kg/min), and walking speed (in m/min) will be determined between the fourth and sixth minute of the test.

### Attendance rate and adherence

The attendance rate (number of sessions followed) for the individualized aerobic exercise and motivational interviewing coaching will be assessed from the ReVi dashboard and logbooks, respectively. Adherence to the aerobic exercise programme will be determined based on time spent in the designated target heart rate zones. Adherence to the coaching sessions will be based on an overall score for MI quality determined by analysing audio recordings of the sessions using the Motivational Interviewing Treatment Integrity (MITI) scoring system [51]. For each practitioner, four audio-recorded sessions of different participants throughout the study will be randomly selected to provide a reliable weighted competency score [52].

### Adverse events

All adverse events reported by participants or observed by therapists will be recorded and followed until they have abated or a stable situation has been reached.

### Data management

Each participant will be randomly assigned a personal identification code (ID), which will be used on all data. All data will be registered in a CASTOR EDC database by direct entry. The participant ID list will be stored with password protection and will only be accessible to the investigators. All files will be kept for 15 years in secure conditions.

### Sample size

We aim to achieve sufficient power to detect differences in both the short term (T1, primary endpoint) and longer term (T2). Because the expected change in VO_2peak_ is somewhat larger at T1, we used change in VO_2peak_ from T0 to T2 for the sample size calculation. Based on previous studies of exercise programmes in NMD, we expect a difference in change in VO_2peak_ from T0 to T2 between the intervention and control group of + 2.5 ml/min/kg (10%) [16, 53, 54].

Based on an effect size of 2.5 ml/min/kg, 1:1 group allocation, standard deviation of 4.7 ml/min/kg (based on previous studies) and a two sided α of 0.05, a sample size of *n* = 76 per group will be needed to obtain 90% power. However, because we will perform an Analysis of Covariance (ANCOVA), in which the baseline measurement will serve as covariate, a correction for the correlation between baseline and follow-up scores should be made [55]. In a previous RCT by our group, the correlation coefficient (r) was 0.71, resulting in *n* = 38 participants per group (76 x (1-r^2^)). As we expect a maximal drop-out rate of 15% based on previous studies [56, 57], 90 participants will be recruited (45 per group).

### Statistical analyses

Data collected in this study are all quantitative and therefore means, medians and percentages (as applicable) will be used as descriptive statistics. Data will be analysed with SPSS statistical software (IBM Corporation, Armonk, NY, USA), and *P* ≤ 0.05 used as significance level. We will perform analyses on intention-to-treat basis and include all randomized participants.

The primary outcome analysis compares the change from baseline to T1 (directly post-intervention) in VO_2peak_ between the groups based on ANCOVA, using the baseline value as covariate. Missing data will be imputed, first by interpolation if possible, and otherwise by multiple imputation. The secondary analysis compares the change from baseline to T2 (12 months follow-up) in VO_2peak_ between the groups based on ANCOVA, using the baseline value and stratification factors as covariates.

Additionally, we will evaluate between-group differences in secondary outcomes at the 6-, 12- and 18-month follow-up assessments (i.e. T1, T2 and T3 respectively), with linear mixed model analysis for repeated measurements. Random effects for the intercept and time will be included in the model. Baseline values, treatment group, time and a group by time interaction term will be included as covariates. In addition, a random effect for treatment centre will be included to account for partial clustering within centres. Multivariate linear regression analysis for longitudinal data will be used to investigate associations between participant and disease characteristics and effect of intervention.

### Withdrawal of participants

Participants can leave the study at any time for any reason if they wish to do so, without any consequences. The investigator can decide to withdraw a participant for urgent medical reasons. Individual participants will not be replaced after withdrawal. Participants who have withdrawn from the intervention will be asked to participate in follow-up measurements.

### Monitoring

Given the low risk for participants, an independent Data Safety and Monitoring Board (DMSB) has not been established. The investigators are responsible for procedures of data monitoring. To facilitate compliance with Good Clinical Practice guidelines, the investigator will permit study-related monitoring, audits, and inspections by authorized organizations.

### Study status

From September 2018 to February 2020, 40 participants were randomized. In the following year we expect to recruit the remaining 50 participants, with the last participant expected to be randomized in March 2021, and finishing the last-follow up measurement in September 2022.

### Patient and public involvement

A multidisciplinary working group consisting of rehabilitation physicians, physical therapists, clinical exercise physiologists and individuals with different NMD were invited to participate in several expert meetings to develop the physical activity program that forms the basis for this I’M FINE project. The expert meetings were used to discuss and adjust the draft versions. Draft versions were developed based on findings of two recent RCTs on aerobic exercise in NMD, experiences of patients and care professionals, and current insights from scientific literature on exercise physiology. A final draft was sent for feedback to the Dutch association for neuromuscular diseases and the Dutch professional associations for rehabilitation medicine and physical therapy. We also incorporated the suggestions from representatives of different diagnoses (CMT and PPS) in the I’M FINE project proposal.

### Public disclosure and publication policy

It is our intention to publish the findings of the study in scientific journals and to present them at scientific meetings. The responsibility for publication and presentation belong to the investigators. Only those investigators making a significant contribution to the study design and/or the collection, analysis or interpretation of the I’M FINE trial data will be eligible for authorship. No restrictions regarding the public disclosure and publication of the research data have been, or will be made, by the funders.

## Discussion

The I’M FINE study will evaluate the efficacy of a six-month physical activity intervention, combining individualized aerobic exercise and motivational interviewing coaching, aimed at improving physical fitness in individuals with slowly progressive NMD in comparison to usual care. The study has several important strengths, which are incorporated in the key components of the I’M FINE intervention and study design.

The individualized aerobic exercise programme was specifically designed for individuals with slowly progressive NMD and is based on polarized protocols, a relatively new type of training. This approach would appear to be better suited to individuals with NMD than conventional training programmes, but has not yet been studied in this population. Furthermore, the prescription of exercise intensity is based on actual fitness levels and therefore better individualized compared to other studies, which generally prescribed intensity based on estimated maximal capacity. Motivational interviewing coaching will be combined with individualized aerobic exercise to support the transition from therapist-supervised exercise to continued physical activity embedded in daily routine. To the best of our knowledge, this is the first RCT in this population that includes an exercise behaviour strategy to enhance the sustainability of intervention effects.

Once completed as envisaged, this study will be the largest RCT of the efficacy of a physical activity programme in NMD ever conducted, with outcomes at all levels of the International Level of Classification (ICF) [58]. This approach will permit detailed evaluation of effects at specific ICF levels and possible interactions. Furthermore, all participants will be followed up for 12 months after the intervention period. A long follow-up period is clinically relevant, but has not been previously investigated in this population. Extended follow-up will not only provide information about the maintenance of health effects and long-term results (e.g. the effects on metabolic syndrome markers), but also about possible long-term adverse events.

Due to the strengths of the I’M FINE intervention, we anticipate lower dropout rates, higher adherence and, consequently, a higher efficacy compared to previously studied physical activity programmes in NMD. Previous studies reported attendance rates, but did generally not report actual adherence to a programme. In this study the use of the specifically designed ReVi app allows detailed monitoring of actual time spent in designated intensity zones. All sessions of the coaching programme will be audio recorded, enabling an in-depth analysis. A potential limitation of this study is the lack of available criteria to quantify the extent to which physical fitness at baseline is reduced due to physical inactivity. Nevertheless, due to study procedures and selection of motivated participants who are not regularly exercising, we expect to recruit a participant group with potential for improvement of physical fitness.

In conclusion, the I’M FINE study will provide evidence regarding the efficacy of a physical activity intervention on the physical fitness and active lifestyle over the short- and long-term in individuals with slowly progressive NMD. These outcomes could potentially improve the (inter)national guidelines for efficacy of aerobic exercise programmes and provide insight in achieving a more active lifestyle in NMD.

## Supplementary information

**Additional file 1:** Appendix 1 - SPIRIT checklist.

## Data Availability

Not applicable.

## Abbreviations

6MPT: 6-min push test
6MWT: 6-min walk test
ACSM: American College of Sports Medicine
ANCOVA: Analysis of Covariance
AT: Anaerobic Threshold
CMT: Charcot-Marie-Tooth disease
FEV1: Forced Expiratory Volume in 1 Second
FVC: Forced Vital Capacity
HDL: High Density Lipoprotein
IC: Inspiratory Capacity
ICF: International Level of Classification
ID: Identification Code
I’M FINE: IMproving FItness in NEuromuscular diseases
LDL: Low Density Lipoprotein
MCS: Mental Health Component Score
MI: Motivational Interviewing
MVT: Maximal Voluntary Torque
MVV: Maximal Voluntary Ventilation
Nm: Newton-meters
NMD: Neuromuscular Diseases
PCS: Physical Health Component Score
PPS: Post-polio syndrome
RCT: Randomized Controlled Trial
ReVi: Rehabilitation and Vitality (*Revalidatie en Vitaal)*
RPM: Revolutions Per Minute
SEPA: Self-Efficacy for Physical Activity scale
SF36: Short Form 36–item Health Survey
T0: baseline assessment
T1: directly post-intervention
T2: 12 months follow-up
T3: 18 months follow-up
VLDL: Very Low Density Lipoprotein
